# Surgical Management of Failed Revascularization in Moyamoya Vasculopathy

**DOI:** 10.3389/fneur.2021.652967

**Published:** 2021-06-29

**Authors:** Kristin Lucia, Güliz Acker, Nicolas Schlinkmann, Stefan Georgiev, Peter Vajkoczy

**Affiliations:** ^1^Department of Neurosurgery, Charité–Universitätsmedizin Berlin (Corporate Member of Freie Universität Berlin, Humboldt–Universität zu Berlin, and Berlin Institute of Health), Berlin, Germany; ^2^Berlin Institute of Health, Berlin, Germany

**Keywords:** moyamoya disease, revascularization, failed revascularization, extra- intracranial bypass, surgical management

## Abstract

**Objectives:** Moyamoya vasculopathy (MMV) is a rare stenoocclusive cerebrovascular disease associated with increased risk of ischemic and hemorrhagic stroke, which can be treated using surgical revascularization techniques. Despite well-established neurosurgical procedures performed in experienced centers, bypass failure associated with neurological symptoms can occur. The current study therefore aims at characterizing the cases of bypass failure and repeat revascularization at a single center.

**Methods:** A single-center retrospective analysis of all patients treated with revascularization surgery for MMV between January 2007 and December 2019 was performed. Angiographic data, cerebral blood flow analysis [H_2_O PET or single-photon emission CT (SPECT)], MRI, and clinical/operative data including follow-up assessments were reviewed.

**Results:** We identified 308 MMV patients with 405 surgically treated hemispheres. Of the 405 hemispheres treated, 15 patients (3.7%) underwent repeat revascularization (median age 38, time to repeat revascularization in 60% of patients was within 1 year of first surgery). The most common cause of repeat revascularization was a symptomatic bypass occlusion (80%). New ischemic lesions were found in 13% of patients prior to repeat revascularization. Persistence of reduced or progressive worsening of cerebrovascular reserve capacity (CVRC) compared with preoperative status was observed in 85% of repeat revascularization cases. Intermediate-flow bypass using a radial artery graft was most commonly used for repeat revascularization (60%) followed by re-superficial temporal artery to middle cerebral artery (re-STA-MCA) bypass (26%). High-flow bypass using a saphenous vein graft and using an occipital artery to MCA bypass was each used once. Following repeat revascularization, no new ischemic events were recorded.

**Conclusion:** Overall, repeat revascularization is needed only in a small percentage of the cases in MMV. A rescue surgery should be considered in those with neurological symptoms and decreased CVRC. Intermediate-flow bypass using a radial artery graft is a reliable technique for patients requiring repeat revascularization. Based on our institutional experience, we propose an algorithm for guiding the decision process in cases of bypass failure.

## Introduction

Moyamoya vasculopathy (MMV) is a rare stenoocclusive cerebrovascular disease affecting the basal cerebral arteries, which leads to the formation of fragile net-like vessels (1). As such, patients with MMV can present with intracranial hemorrhage from abnormal vessels as well as ischemic events (2, 3). To date, surgical revascularization remains the primary treatment for MMV, as it aims at restoring perfusion in order to stabilize cerebrovascular hemodynamics and reduce hemorrhagic and ischemic events (4, 5). Three primary surgical strategies can be utilized in the treatment of MMV: direct, indirect, and combined revascularization.

In the case of direct revascularization, anastomosis is performed between branches of the internal and external carotid arteries (ECAs). Most commonly, the superficial temporal artery (STA) is anastomosed with the middle cerebral artery (MCA) (STA-MCA bypass) (6). The anterior and posterior cerebral arteries (ACAs and PCAs) as recipient vessels or the occipital artery (OA) as a donor vessel may also be used to adapt revascularization to territories where it is most decreased (7). Indirect revascularization relies on neoangiogenesis between the cortical surface and a pedicled graft such as the dura mater [encephalodurosynangiosis (EDS)] or the temporalis muscle [encephalomyosynangiosis (EMS)] (8). Direct and indirect revascularization techniques can also be combined in an attempt to improve hemodynamic response over time (for example, STA-MCA bypass plus EDS or EMS) (9, 10). The decision to implement a particular bypass strategy for initial revascularization is made on an individual basis under consideration of patient risk profiles and the expected efficacy. To date, evidence points toward the use of direct or combined revascularization procedures (6–9).

Despite careful planning and initially successful surgical intervention with a high early bypass patency rate of up to 99% in MMV, some patients may continue to develop clinical symptoms of hemodynamic insufficiency (10, 11). Several diagnostic tools may be implemented to evaluate bypass insufficiency including angiographic studies; H_2_O PET for analysis of cerebrovascular reserve capacity (CVRC) and detailed clinical assessment of the severity of the symptoms were described (12). Failed bypass surgery can be traced back to technical failures such as low flow delivery or secondary occlusion of a direct graft, which failed to take an indirect graft or insufficient collateralization. Disease progression in new vascular territories (i.e., posterior circulation) may also necessitate implementing a rescue strategy for revascularization. Whereas, selection of an appropriate rescue strategy should take the underlying cause of initial bypass failure into consideration, several techniques of direct or combined revascularization have been described to date as useful rescue strategies. These include radial artery grafts (radial artery grafts) from the ECA to the M2 or M3 segment of the MCA (RAG-ECA-MCA) (11), direct bypass of the OA to the PCA (OA-PCA) (13), or repeat STA-MCA bypass using an alternative branch of the STA (14).

Considering the variability of indications and surgical strategies for rescue revascularization in failed bypass surgery, we aimed at identifying the cases of repeat revascularization in our department in order to further characterize the causes of bypass failure, the indications for repeat surgery, and the surgical strategies that were implemented.

## Materials and Methods

### Study Design

This retrospective analysis was approved by the local ethics committee and was conducted in accordance with the Declaration of Helsinki.

We retrospectively identified all moyamoya patients who were treated for failed revascularization in our department between January 2007 and December 2019 including those with unilateral moyamoya and moyamoya syndrome by using the International Classification of Diseases (ICD) code 167.5 (moyamoya). Moyamoya disease (MMD), moyamoya syndrome, and unilateral MMV were defined based on the guidelines established by the Research Committee for Moyamoya Disease; and patients with possible associated diseases were classified as moyamoya syndrome patients (2). Electronic medical records were used to gather data on patient demographics; time and type of initial surgery; follow-up time; angiographic, MRI, and hemodynamic diagnostics; and clinical outcome.

### Statistical Analysis

Quantitative values are presented as median values with range. Group comparisons were performed using Fischer's exact test. Univariate Cox regression analysis was performed among all cases of surgically treated MMV using repeat revascularization as an outcome event. Multivariate regression analysis was conducted using repeat revascularization as a dependent variable when the univariate analysis delivered a *p* < 0.015. *p* < 0.05 were considered statistically significant. All analyses were done with Excel (version 14.7.7; Microsoft) and SPSS (version 24; IBM Corp.).

## Results

### Patient Characteristics

We analyzed a total of 405 surgically treated hemispheres in 308 MMV patients. Of these patients, 68.5% had MMD, 8.1% had unilateral MMD (uMMD), and 25% had moyamoya syndrome. In our series, 19% of patients were juvenile. The median age at first surgical intervention was 38 years (range 2–63 years old, Table 1). Seventy-five percent of patients initially presented with ischemic events, and the remaining 25% presented with hemorrhagic lesions. Regarding gender distribution, 28% of patients were male and 72% were female. The majority of our cohort were of Caucasian background (87%), 8% were of Asian descent, and 5% were Middle Eastern descent.

**Table 1:** Demographics and disease characteristics in revision cases and total cases.

	**Revision**	**Non-revision**	***p***
**Total**	15 (3.7%)	390	0.35
Age in years (median/range)	38/10–57	33/2–63	0.23
Suzuki Grade 1 2 3 4 5	*N* = 15 3 (20%) 3 (20%) 3 (20%) 3 (20%) 3 (20%)	*N* = 390 78 (20%) 78 (20%) 78 (20%) 50 (13%) 106 (27%)	1.000 1.000 1.000 0.440 0.533
Berlin Grade 1 2 3	*N* = 15 1 (7%) 3 (20%) 11 (73%)	*N* = 206 26 (13%) 62 (30%) 118 (57%)	0.705 0.569 0.178

*Suzuki Classification was performed for all hemispheres. Data on Berlin Classification was available for 206 of 405 hemispheres (51%). Fischer's Exact Test. p < 0.05 is considered significant*.

A total of 20% of hemispheres each were classified as Suzuki stages 1, 2, and 3. Thirteen percent were Suzuki stage 4, and 27% were Suzuki stage 5 (Table 1). The Berlin Grading (15) could be calculated based on complete data available for 206 hemispheres (51%). Of these hemispheres, 13% of hemispheres were grade 1, 30% were grade 2, and 57% were grade 3.

Cardiovascular comorbidities including hyperlipidemia and hypertension were found in 40 and 26% of patients requiring repeat revascularization, respectively.

### First Revascularization Strategies

The most common initial revascularization strategy was STA-MCA bypass combined with EDS (44%), followed by STA-MCA bypass alone (20%) (Table 2). The median follow-up period after revascularization in unilateral cases was 12.7 months (range 3–47 months). In bilateral cases, median follow-up for the first hemisphere was 12.8 months (range 1–79 months) and for the second hemisphere 7.7 months (range 1–35 months). A total of 29 hemispheres did not receive follow-up on site after the revascularization (10 unilateral and 19 bilateral cases for the second hemisphere treated). Nine of these patients had traveled from abroad for surgery in our department and received a follow-up care in their home countries.

**Table 2:** Initial symptoms and surgical strategies for initial revascularization.

	**Revision** **cases (*n =* 15)**	**Remaining** **Cases** **(*n =* 293)**	***p***
**Initial symptoms**			
Ischemia	13 (87%)	231 (79%)	0.719
Hemorrhage	2 (13%)	62 (21%)	0.216
	**Revision cases** **(*****n=*** **15)**	**Remaining cases (*****n=*** **390)**	***p***
**First surgical strategy**			
STA/MCA + EDS	11 (73%)	173 (44%)	0.251
STA/MCA + EMS	2 (13%)	67 (17%)	0.329
STA/MCA + EDS/EMS	1 (67%)	50 (13%)	0.158
ECA/MCA	1 (67%)	4 (1%)	0.441
STA-MCA alone	0	78 (20%	–
EDGS	0	4 (1%)	–
EDS or EMS alone	0	14 (4%)	–

*Calculations of initial symptoms are based on total patients (n = 308, 15 revision cases and 293 remaining) and surgical strategies on total hemispheres (n = 405). Fischer's exact test. p < 0.05 is considered significant*.

*STA-MCA, superficial temporal artery to middle cerebral artery; EDS, encephalodurosynangiosis; EMS, encephalomyosynangiosis; EDGS, Encephalodurogaleosynangiosis*.

### Demographics and Surgical Strategies for Initial Revascularization in Failed Cases

The most common initial surgical strategy used in patients was an STA-MCA bypass combined with EDS (73%). Of these cases, only 15 hemispheres (one hemisphere per patient) required surgical revision (3.7%). The median age of patients undergoing surgical revision was 38 years (range 10–57 years). A total of 40% of patients (6/15) received revision surgery within 1 year following the initial procedure. The majority of patients were female (13/15, 87%). The majority of the patients were diagnosed with MMD (12/15, 80%) with only 20% quasi-MMD. Most patients initially presented with ischemic symptoms before their initial surgery (12/15, 80%). The operated hemisphere in patients requiring revision surgery represented with 20% each of Suzuki grades 1–5. With the use of the Berlin Grading system, one hemisphere was grade 1 (6.6%), three hemispheres were grade 2 (20%), and 11 were grade 3 (73.3%).

Regarding the first surgical strategy, 11/15 patients (73%) had been treated with a combined revascularization strategy using STA-MCA bypass and EDS and 2/15 (13%) with STA-MCA bypass and EMS. One patient (6.7%) was treated with combined STA-MCA plus EDS and EMS; and one patient received initial revascularization through an ECA-MCA bypass with radial artery graft. No statistical difference could be found in the proportions of initial surgical strategies used in revision cases vs. non-revision cases (Table 2).

### Indications for Repeat Revascularization and Surgical Strategies

The most common cause of surgical revision was ischemic symptoms corresponding to bypass occlusion on digital subtraction angiography (DSA) (found in 12 of 15 cases, 80%). Of the remaining three cases, one procedure was performed in a symptomatic patient with a patent bypass, and one case was repeated due to secondary occlusion of the A. cerebri posterior (P2 segment). In a further case, the initial revascularization procedure had to be ended intraoperatively due to severe vasospasm (Table 3).

**Table 3:** Surgical strategies, indication, and timing of repeat revascularization.

**Second surgical strategy**	
STA/MCA (frontal branch)	4 (26%)
ECA/MCA (RAG interposition)	9 (60%)
ECA/MCA (SV interposition)	1 (67%)
OA/MCA	1 (67%)
**Reason for revision**	
Symptomatic bypass occlusion on DSA	12 (80%)
Symptomatic patent bypass	1 (67%)
Intraoperative vasospasm (surgery not completed)	1 (67%)
Secondary P2 occlusion	1 (67%)
**Time between initial surgery and revision**	
0–1 Year	6 (40%)
1–3 Years	3 (20%)
3–6 Years	3 (20%)
>6 Years	3 (20%)

*Summary of second surgical strategies, reason for repeat revascularization, and time between initial surgery and repeat revascularization*.

*STA-MCA, superficial temporal artery to middle cerebral artery; ECA-MCA, external carotid artery to middle cerebral artery; RAG, radial artery graft; SV, saphenous vein; OA-MCA, occipital artery to middle cerebral artery*.

The most common symptom prior to repeat revascularization was transient ischemic attacks (TIAs) (8/15, 53%) followed by new paresthesias (4/15, 27%). One patient suffered a new hemorrhage prior to repeat revascularization, and one suffered new epileptic seizures (Table 4).

**Table 4:** Imaging and clinical characteristics before and after repeat revascularization.

	**Before revision**	**After revision** **(last follow-up)**
**CVRC**		
Unchanged to preoperative status	11 (73%)	9 (60%)
Worsened to preoperative status	1 (67%)	0
Improved to preoperative status	0	2 (13%)
Data not available	2 (13%)	2 (13%)
**New ischemic lesions**		
Yes	2 (13%)	0
No	13 (87%)	15
**Symptoms**		
TIAs	6 (40%)	0
Hemorrhage	1 (67%)	0
Persisting paraestheisa	4 (33%)	0
Epileptic seizures	1 (6 7%)	0
Stable/No new clinical symptoms	1 (67%)	10 (66%)
Improved clinical status	0	5 (33%)

*Summary of cerebral blood flow measurements (H_2_O PET or SPECT), ischemic lesions on MRI, and neurological symptoms before and after repeat revascularization*.

*CVRC, cerebrovascular reserve capacity; TIA, transient ischemic attack; SPECT, single-photon emission CT*.

We also examined the CVRC as measured by PET or single-photon emission CT (SPECT) before surgical revision, compared with preoperative status. In two cases (13%), no additional measurement was performed before revision surgery (one case due to thrombus of the bypass after discontinuing ASS and one case following intraoperative vasospasm). In 85% of cases (11/13), the CVRC before surgical revision was unchanged to the status before the initial surgery, and two patients (15%) displayed worsening CVRC (Table 4).

**Figure 1:**
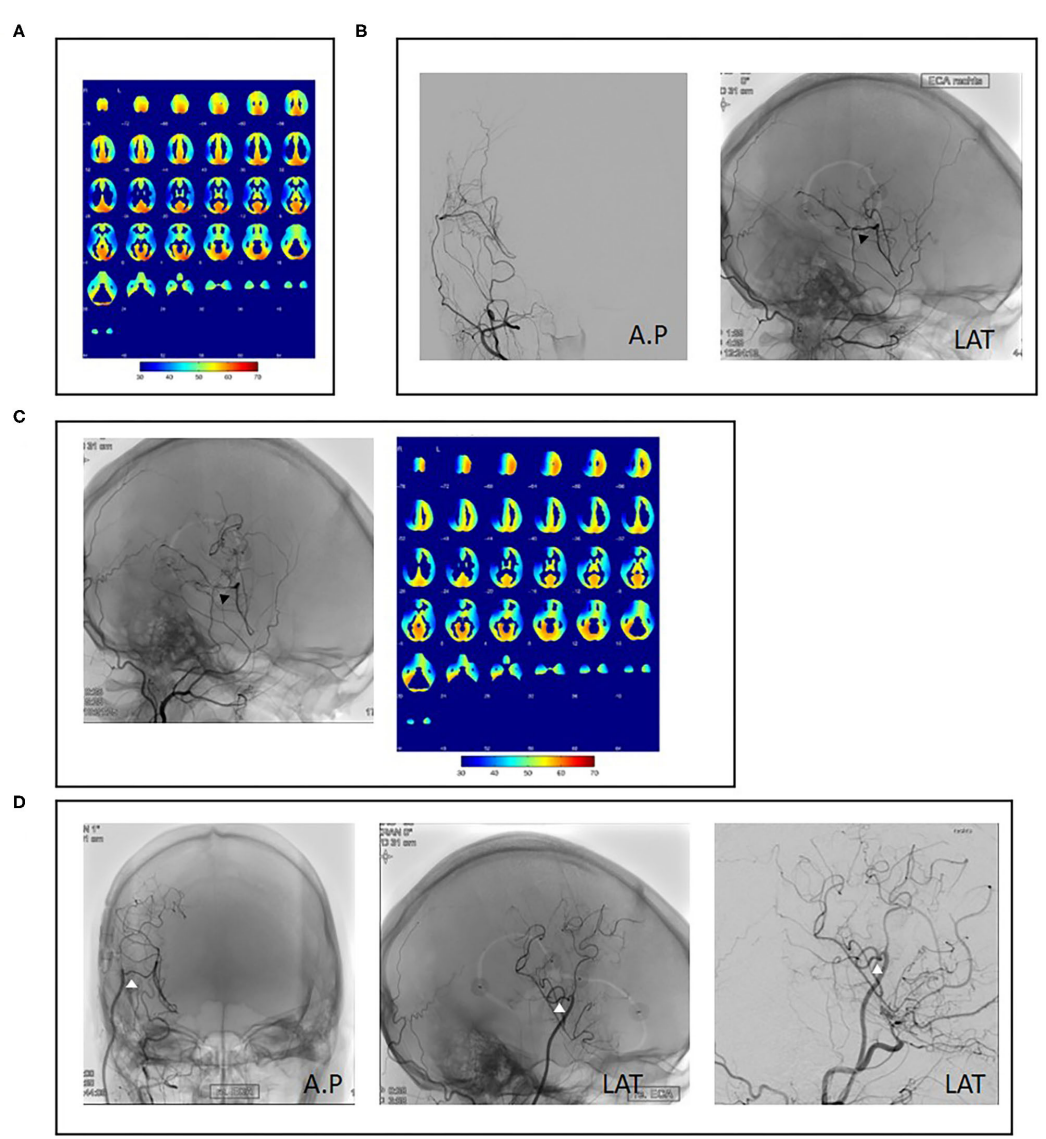
Case example of repeat revascularization using a radial artery graft. A 34-year-old female patient presented with right hemispheric transient ischemic attacks (TIAs) and angiographic evidence of unilateral right-sided moyamoya disease as well as decreased cerebrovascular reserve capacity (CVRC) of the right hemisphere **(A)**. Revascularization using superficial temporal artery to middle cerebral artery (STA-MCA) plus encephalodurosynangiosis (EDS) was performed without complication (**B**, black arrowheads). Fourteen months after initial surgery, the patient reported new left-sided hemihypesthesia. Angiography showed decreased bypass patency and decreased CVRC of the anterior MCA territory (**C**, black arrowheads). As no frontal STA branch was available, repeat revascularization was performed with a radial artery graft (**D**, white arrowheads).

**Figure 2:**
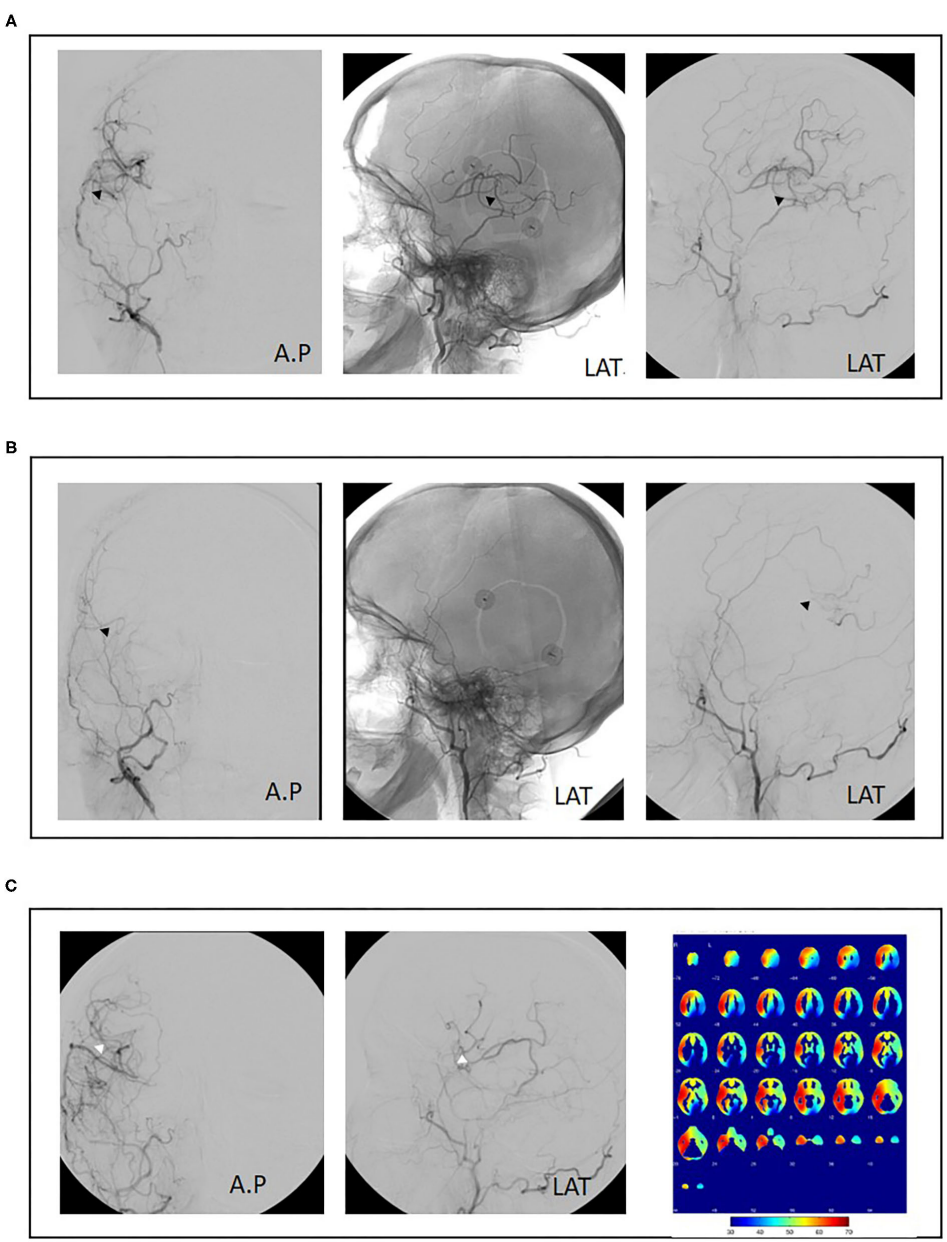
Case example of repeat revascularization using superficial temporal artery to middle cerebral artery (re-STA-MCA). A 50-year-old female patient with bilateral moyamoya disease and right hemispheric transient ischemic attacks (TIAs) was treated with combined revascularization using STA-MCA (parietal branch) plus encephalodurosynangiosis (EDS) of the right hemisphere (**A**, black arrowheads). Postoperatively, no new neurological symptoms were observed. Eight months postoperatively, the patient suffered a fractured radius requiring orthopedic surgery. Aspirin was discontinued preoperatively and not resumed after surgery. The patient then presented with renewed right-sided TIAs. Angiography showed bypass occlusion (**B**, black arrowheads). Repeat revascularization was performed using a frontal branch of the STA plus EDS. Cerebral blood flow analysis 1 year after initial surgery shows adequate perfusion of the right hemisphere. Revascularization of the left hemisphere was performed consecutively (**C**, white arrowheads).

The surgical revision strategy most commonly employed was the ECA-MCA using a radial artery graft (9/15, 60%) (Figure 1). In one case, the ECA-MCA bypass was completed using a saphenous vein (SV) graft due to hereditary systemic sclerosis of arterial vessels. Four cases (26%) were performed using a frontal STA branch (Figures 2A–C). One case was performed using an OA-MCA bypass (Table 3).

### Follow-Up After Repeat Revascularization and Preoperative Predictors of Bypass Failure

The median observational period following repeat revascularization was 12 months (range 3–60 months). Following repeat revascularization, 10/15 patients (66%) maintained a stable neurological status and developed no new clinical symptoms, whereas 5/10 patients (33%) reported improved clinical status compared with before the repeat revascularization (Table 4). CVRC measurements were performed in 11 of 15 patients following repeat revascularization between 1 and 5 years after last surgery. Here, 82% (9/11) of patients showed unchanged CVRC, with 18% (2/11) having improved CVRC compared with preoperative status before the first revascularization attempt, and no patients showed worsening of CVRC following revision surgery. In 13% of patients (2/15), new ischemic lesions could be detected on MRI before repeat revascularization. No new ischemic lesions could be found at last observation in patients following revision surgery (Table 4).

We performed univariate Cox regression analysis to model the relationship of preoperative patient characteristics with the event of repeat revascularization considering gender, age (adult vs. child), ethnic background, symptoms at initial presentation, ischemic lesions on MRI, reduced CVRC, and Berlin Grading as independent variables. Univariate Cox regression analysis showed no significant increase in the proportional hazard ratio of the above-mentioned predictors on the event of repeat revascularization (Table 5).

**Table 5:** Model summary of univariate Cox regression analysis.

**Predictor**	**Hazard** **ratio**	**CI (95%)**	***P***
Gender	0.944	0.614–1.455	0.796
Age (Adult vs. Child)	1.173	0.829–1.659	0.367
Ethnicity (Caucasian vs. Asian)	1.016	0.624–1.656	0.948
Clinical Presentation (Ischemia vs. Hemorrhage)	0.716	0.455–1.125	0.147
Ischemia on MRI	1.360	0.970–1.907	0.074
CVRC (Reduced vs. Preserved)	0.819	0.823–1.383	0.385
Berlin grade	0.917	0.346–2.433	0.863

*CVRC, cerebrovascular reserve capacity*.

Subsequent multivariate analysis included the variables “ischemic lesions on MRI prior to first surgery” as well as “symptoms at initial presentation.” This analysis however displayed no significant relationship with the event of repeat revascularization, whereas “ischemic lesions” nearly reached significance at *p* = 0.054 (Table 6).

**Table 6:** Model summary of multivariate Cox regression analysis.

**Predictor**	**Hazard** **ratio**	**CI (95%)**	***P***
Clinical presentation (Ischemia vs. Hemorrhage)	1.461	0.000–4.667	0.965
Ischemia on MRI	0.301	0.089–1.020	0.054

## Discussion

In this study, we reviewed our institutional series of cases requiring repeat revascularization in patients with MMV. We observed a low rate of bypass failure with only 3.7% in our patient cohort with good outcome after rescue operation. We did not identify any significant predictors for the bypass failure. The clear predominance of female patients in our series may be attributed to the overall female predominance in sporadic and familial MMD, especially in Caucasian patients (3, 25, 26). In contrast to previous series on repeat revascularization in which re-STA-MCA was most commonly utilized (17), the most commonly implemented technique in our cohort was the intermediate-flow bypass using a radial artery graft.

Our analysis of 405 operated hemispheres showed an overall rate of repeat revascularization due to bypass failure of 3.7% similar to the previous large North American institutional series with 4% (14). In our previous analysis, the failure rate among 122 patients was similar at 3.2% (11). While there are very few studies specifically focusing on moyamoya patients requiring repeat revascularization surgery, examining data on the rate of radiological STA-MCA bypass patency for MMV showed an early bypass patency rate of up to 99% at 1.5 years in the series of Guzman et al. (10), which is similar to the early bypass patency for other indications than moyamoya with up to 96% (16–22). Schick et al. reported a late graft failure (between 1.4 and 2.7 years following initial surgery) of approximately 10% in non-MMV patients within 5.6 years of follow-up (17). In our MMS cohort of 61 patients, the short- and long-term bypass patency was similar with 93 and 88% (within a mean follow-up period of 4.2 years), respectively (23). Importantly, a bypass occlusion does not always require a revision. A further theory regarding the etiology of bypass occlusion has pointed to a possible complementary role of indirect revascularization when combined with direct revascularization. Here, it has been proposed that the collaterals formed by indirect revascularization techniques may abrogate the demand placed on direct grafts, therefore allowing for their successive occlusion after combined procedures (16). However, in these cases, patients do not present with new symptoms and have maintained CVRC. Furthermore, the possible role of cardiovascular comorbidities in promoting bypass occlusion also has yet to be explored. The rates of hyperlipidemia (40%) and hypertension (26%) in our cohort of revised bypasses appear to differ from those of a previously reported German cohort including all entities of moyamoya (hyperlipidemia 15% and hypertension 50%) (24). Further analysis of whether or not these differences are pathophysiologically relevant or due to differences in cohort size and selection of bypass failure is necessary.

The lower proportion of patients requiring surgical intervention may underscore the importance of additional analysis of CVRC and clinical symptoms when considering a second surgical intervention. The review of our cases showed that iatrogenic acute graft occlusion or intraoperative complications account for only a minority of repeat revascularizations (2/15, 13%). One such case was due to symptomatic bypass occlusion during prolonged discontinuation of antiplatelet therapy following elective orthopedic surgery; the other was due to intraoperative vasospasm (in this case, the procedure was completed in a second session without complication). In the series of Teo et al. one patient underwent revascularization as a result of acute graft occlusion within 1 week following initial surgery (14). The remaining 12 cases (80%) in our series were performed due to symptomatic bypass occlusion. All patients showed reduced CVRC under acetazolamide challenge in H_2_O PET or SPECT. In contrast, Teo et al. described only one case of graft occlusion following direct revascularization (STA-MCA), although 24/29 (83%) patients for whom cerebral blood flow studies were available showed reduced CVRC under acetazolamide challenge (14). These findings may indicate disease progression as a driver for repeat revascularization surgery in this cohort, whereas our series primarily received revascularization under conditions of correlation between bypass occlusion, neurological symptoms, and reduced CVRC and not bypass occlusion alone.

We did not identify any significant predictors for bypass failure; however, ischemic lesions in MRI prior to surgery seem to be the most relevant factor (*p* = 0.054). The Berlin classification was shown to correlate with symptoms and perioperative complications (15, 25, 26); however, it did not predict failed revascularization in our cohort most probably due to the low number of revision cases in our series. The majority of the revised cases (73%) were grade 3 according to the Berlin classification, indicating the severity of the disease in these patients.

Regarding the time points at which revascularization was performed, we found no clear distribution in our series. While 6/15 (40%) of patients underwent repeat revascularization within 1 year following initial intervention, this number includes the two unusual cases of discontinued antiplatelet therapy and intraoperative vasospasm. Observing these numbers without these two cases shows a nearly equal distribution of repeat revascularization at all time points (within 1 year after surgery; 1–3, 3–6, and over 6 years after surgery). These findings differ from those of Teo et al. in which the mean time of repeat revascularization surgery was 47 months (14). This discrepancy may be due to the difference of case numbers, as our series includes 15 (of 405) vs. 57 (of 1244) hemispheres in the North American series. An additional study regarding STA-MCA graft failure performed in patients suffering from atherosclerotic occlusion of the internal carotid artery bypass occlusion has shown rates of bypass failure in up to 10% of patients at an average of 38 months following initial surgery (17).

In our cohort, all patients were initially treated with direct or combined revascularization techniques. In contrast to our cohort in which only one patient (6.7%) showed new P2 stenoocclusion, the majority of patients in the North American series (11/20, 55%) received repeat surgery due to angiographic evidence of poor filling of another vascular territory and most commonly in the anterior MCA territory (14). This discrepancy may be due to the diagnostic criteria utilized to determine the indication for repeat vascularization (angiographic findings vs. cerebral blood flow studies). Regardless, these findings point toward the necessity of further characterization of disease progression in other vascular territories aside from the MCA, which is most commonly targeted by standard direct revascularization. Deciphering cases of insufficient leptomeningeal collateralization following MCA revascularization from separate progression of ACA-PCA insufficiency may help guide the choice of revascularization technique (re-STA-MCA vs. STA-ACA or OA-MCA).

In our study, no fatalities in relation to repeat revascularization were observed. The use of a high-flow SV graft as a repeat revascularization strategy has been previously reported to have led to fatal reperfusion hemorrhage in one case (14). Our series also included one patient who received SV graft under strict perioperative normotensive therapy without complications. In our experience, the SV graft when performed under consistent perioperative blood pressure monitoring and maintenance of normotension is a viable strategy and has been shown to demonstrate long-term patency (27).

**Figure 3:**
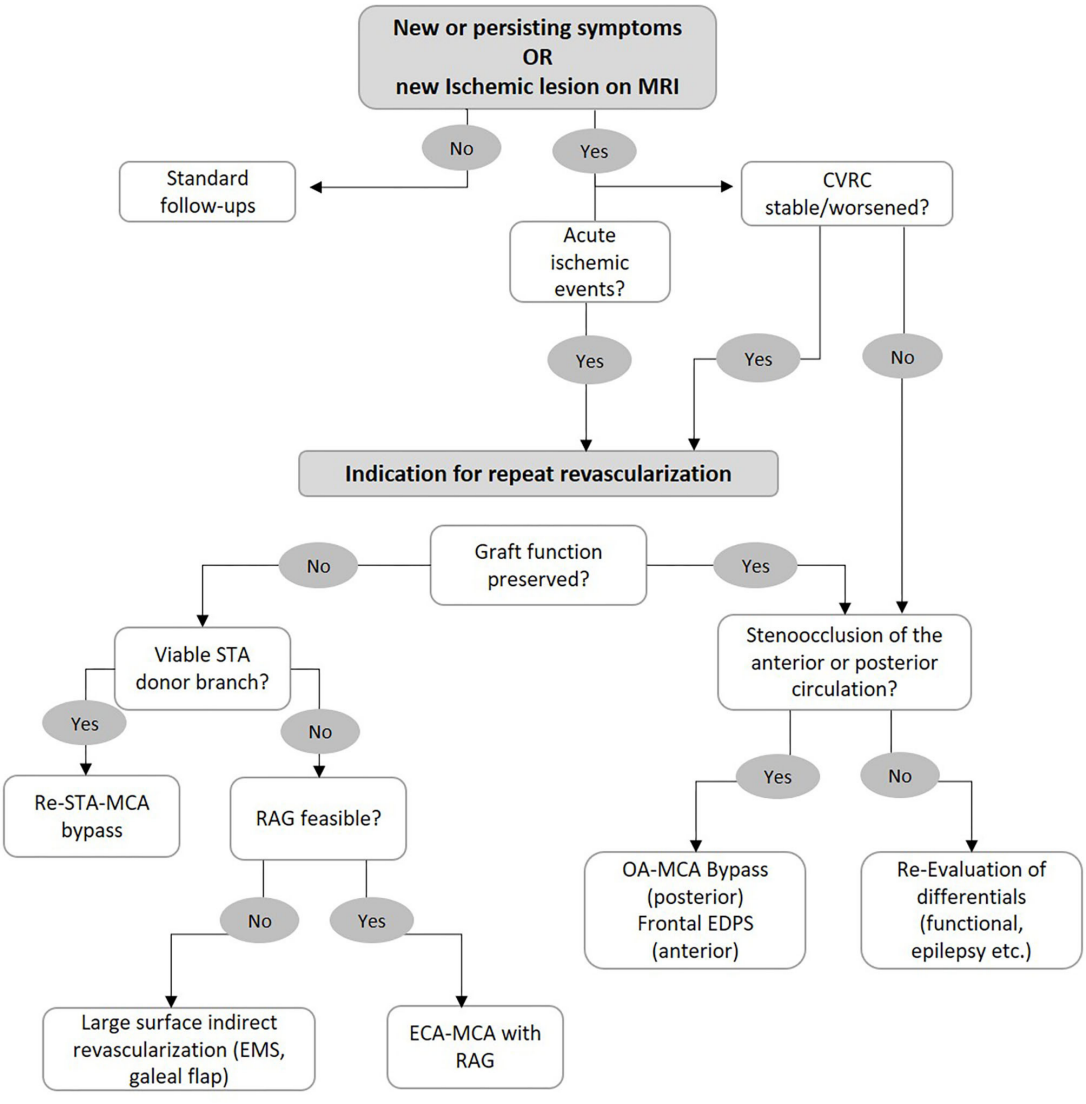
Flowchart for selection of surgical strategy in case of bypass failure. See main text for further information.

Patients who are determined to require repeat revascularization surgery are therefore identified based on the presence of clinical symptoms in addition to reduced CVRC as measured by H_2_O PET or SPECT under acetazolamide challenge. Based on our institutional experience, we have developed an algorithm to aid in our selection of the surgical strategy for patients with an indication for repeat revascularization surgery (Figure 3). Repeat STA-MCA bypass plus EMS or EDS is performed if a second branch of the STA is available for use in a re-STA-MCA, and the reduced CVRC can be adequately addressed with this bypass. We prefer a combined revascularization, as previous studies have shown the superiority of combined vs. indirect revascularization regarding hemodynamic normalization as well as the rate of secondary ischemic events (4, 6, 8). If no donor branch is available, the patient is assessed for feasibility of a RAG. This includes sonographic measurement of arterial diameter, flow, and functional determination of adequate collateral circulation of the hand (positive Allen's test). The presence of atherosclerosis of the ECA, stents, or other variables affecting its patency must also be assessed. We have already shown the efficacy and safety of RAG for rescue revascularization (11). If a RAG bypass is determined to not be safely feasible, large surface indirect revascularization (i.e., EMS and galeal flap) and high-flow grafts using the SV remain as alternative strategies.

If a patient meets the criteria for repeat revascularization although the graft patency and function in angiographic series is not found to be insufficient, we examine the progression of the posterior circulation. If new P2 stenoocclusive changes are found, we recommend an OA-MCA bypass. If the anterior circulation is affected and the ACA shows hypoperfusion, then frontal encephalo-duro-periosteal-synangiosis (EDPS) may be performed. If no new steno-occlusion is found in these patients, we recommend re-evaluation and consideration of differentials of clinical status such as functional lesions or epileptic seizures.

The major limitation of the current study is the retrospective design performed in a single institution; however, considering the rarity of MMV in Europe, this is the largest European series analyzed in respect to repeat revascularization. Furthermore, by conducting a time-to-event analysis in a retrospective cohort, possible modifiers associated with the necessity of bypass revision may not have been accounted for.

In conclusion, our study confirms the efficacy of the revascularization surgery in MMV with only a low rate of rescue revascularization. Nevertheless, we suggest the continuing review and critical analysis of failed bypass surgery over time in order to identify trends that may become visible as case numbers increase. Furthermore, examination of non-surgical factors such as relevant cardiovascular comorbidities or biochemical parameters should be considered. Ultimately, the practice of reviewing and analyzing failed revascularization cases should be considered an essential part of the ongoing management of patients with MMV.

## Data Availability Statement

The raw data supporting the conclusions of this article will be made available by the authors, without undue reservation.

## Ethics Statement

The studies involving human participants were reviewed and approved by Charité Universitätsmedizin Berlin. The patients/participants provided their written informed consent to participate in this study. Written informed consent was obtained from the individual(s) for the publication of any potentially identifiable images or data included in this article.

## Author Contributions

KL, GA, and PV conceptualized the study. NS and SG collected patient data. KL and GA analyzed the data and wrote the manuscript including figure and table production. PV reviewed and approved the final manuscript. All authors contributed to the manuscript and approved the final submitted version.

## Conflict of Interest

The authors declare that the research was conducted in the absence of any commercial or financial relationships that could be construed as a potential conflict of interest.
